# Determination and Characterization of Gold Nanoparticles in Liquor Using Asymmetric Flow Field-Flow Fractionation Hyphenated with Inductively Coupled Plasma Mass Spectrometry

**DOI:** 10.3390/molecules29010248

**Published:** 2024-01-03

**Authors:** Bin Li, Sew Lay Chua, Dingyi Yu, Sheot Harn Chan, Angela Li

**Affiliations:** National Centre for Food Science, Singapore Food Agency, 7 International Business Park, Singapore 609919, Singapore; li_bin@sfa.gov.sg (B.L.); chua_sew_lay@sfa.gov.sg (S.L.C.); chan_sheot_harn@sfa.gov.sg (S.H.C.); angela_li@sfa.gov.sg (A.L.)

**Keywords:** gold nanoparticles, ultrasound, gold flakes, gold-containing liquor, AF4-ICP-MS, food additive

## Abstract

The EU has approved the usage of gold as a food additive (E175) and it has been applied in numerous foods for coloring and decoration purposes. Different from the general assumption that edible gold is mainly present in the form of flakes or external coating in foods, this work demonstrated that gold nanoparticles (Au NPs) can be released from gold flakes and extracted under optimized conditions. To support future risk assessment associated with the exposure of Au NPs to human health, an effective approach was established in this study for both size characterization and mass determination of Au NPs released in a commercial gold-containing liquor using Asymmetric Flow Field-flow Fractionation (AF4) hyphenated with Inductively Coupled Plasma Mass Spectrometry (ICP-MS). Our results showed that no Au NPs were detected in the original liquor product and only after ultrasonication for several minutes did Au NPs occur in the ultrasound-treated liquor. Particularly, Au NPs released in the liquor can be well extracted after 100-fold enrichment of gold flakes and the subsequent ultrasonication for 25 min. Size characterization of Au NPs was conducted by AF4-ICP-MS under calibration with Au NP standards. The gold particle sizes detected ranged from 8.3–398.0 nm and the dominant size of the released Au NPs was around 123.7 nm in the processed liquor. The mass concentration of gold particles determined in the liquor sample with gold flakes concentrated and subsequently sonicated was 48.1 μg L^−1^ by pre-channel calibration and the overall detection recoveries ranged over 82–95%. For the comparison control samples without ultrasonication, there was no detection of Au NPs. The established method was demonstrated to be useful for monitoring Au NPs in liquor and is possibly applied to other similar foodstuffs.

## 1. Introduction

Edible gold (Au) has been approved by the EU as a food additive with the code E175 and used in many food products, such as for the decoration of liquors and chocolates, for the coloring or coating of confectioneries and cake sprinkles, etc. [1]. Since the gold flakes or powders are generally used in E175 with a particle size of not less than 1 mm [2], they are commonly considered to go through the human gastrointestinal tract and be excreted without extensive absorption and reaction in digestive juices (e.g., gastric acid) [3]; hence, they are generally deemed quite safe because gold in bulk form is chemically inert and presents no toxicity to human health if it is not ionized. In this regard, the main health risk is generally expected to come from micro-sized or even bigger particles of gold flakes which may be easily trapped in the human digestive organs for quite a while and may cause chronic inflammation or even carcinogenesis in the digestive organs [3]. Additionally, it was also reported that allergic reactions and lichen planus could also be induced by edible gold [4,5,6], which was suspected to be relevant to the dissolved ionic gold from the metallic flakes [1,7,8]. However, as a consequence of the manufacturing process, gold flakes or powders in food-grade gold are expected to exhibit sub-micro- and/or nanometric dimensions in their thickness. It is thus rationally expected that minute amounts of gold nanoparticles (Au NPs) are potentially present in E175 [1,9]. Also, there is the possibility that metallic particles/particulates can be generated with the application of ultrasound processing [10]. In general, ultrasound is a process of converting sound energy into physical vibrations. Although it has been extensively applied to various fields and numerous food and beverage products in the food industry today [11,12,13,14,15], the improper application of ultrasound may break large granules into smaller pieces/fragments, imposing potential contaminations on food products. Consequently, concerns have been raised about the risks to human health associated with the Au NPs generated at low abundance in edible gold flakes and gold-containing foods during their manufacturing.

Historically, although elemental gold (E175) was evaluated by EFSA and had been approved in the EU as a legal food additive, no acceptable daily intake (ADI) of E175 had been established since there was a lack of sufficient data on its toxicity for the risk assessment [1]. Even so, EFSA still recommends that when E175 is used, its specifications must be included, such as the mean particle size, the particle size distribution, and the percentage of particles at the nanoscale [1]. In contrast, the FDA has not approved the use of gold as a food additive and there even has not been a statement on edible gold issued by this authority [2]. In view of the various toxicities or hazardous risks of Au NPs on human health in previous reports [16,17,18] and the lack of a standardized analytical approach, the effective and practical method for characterization and quantification of Au NPs in foodstuffs is thus necessary for the risk assessment of Au NPs in E175 as a safe food additive. To achieve this target, commercial gold-containing liquor was selected as the initial model for Au NP analysis. This arose from the concerns, including (1) the affirmatory cases of patients consuming gold-containing liquor reported previously [4,5,6] and (2) the popularity of gold-containing liquor on the market.

As previously reported, many techniques have been applied to characterize Au NPs, e.g., transmission electron microscopy (TEM) [19,20], single-particle inductively coupled plasma mass spectroscopy (spICP-MS) [21,22,23], dynamic light scattering (DLS) [24,25], etc. Among these techniques, spICP-MS is especially powerful in simultaneously counting and sizing particles with high sensitivity even without complex sample processing when relatively simple sample matrices are involved [21]. However, it is also worth noting that certain inherent features in spICP-MS may narrow down the detectable range of Au NPs as analytes, such as the relatively high size detection limit of Au NPs by spICP-MS (e.g., 13–15 nm as previously reported [26]), the susceptible interferences from signals of the dissolved (ionic) element on the nanoparticles detection especially for analytes at trace concentration. In addition, since there had been reports that the aggregation of Au NPs could be observed in ethanol [27,28], size characterization, measurement of the particle size distribution (PSD), and mass content of the highly polydisperse analytes as minute and aggregated Au NPs represented challenging for these common methodologies. Considering the relatively high concentration of ethanol in most of the commercial liquor products (e.g., ~30–60% (*v*/*v*)), the potentially occurring aggregates of Au NPs in liquor due to effects from ethanol had to be explored and characterized. In this regard, Asymmetric Flow Field-flow Fractionation hyphenated with Inductively Coupled Plasma Mass Spectrometry (AF4-ICP-MS) emerged as a powerful technique for the analysis of Au NPs in this scenario. For the AF4-ICP-MS analysis, fractionation can be achieved from the separation of Au NPs on an ultrafiltration membrane under the cross-flow field. Thus, AF4 is particularly suitable for the size differentiation and enrichment of Au NPs with highly polydisperse aggregates at low abundance. In fact, AF4-ICP-MS has been extensively utilized in the characterization of Au NPs [29,30,31,32] regarding their size and mass fractions.

This study aims to develop an effective and practical AF4-ICP-MS method for both size characterization and mass determination of Au NPs released in commercial gold-containing liquor, with certain novelties demonstrated. One new aspect of this work is the investigation of the effects of alcoholic matrix-like liquor on Au NPs together with the extraction of Au NPs from gold flakes in the liquor product. The established sample preparation method ensures the advantages in terms of sensitivity, simplicity, and compatibility with AF4 separation. Another innovative strength of this work lies in the simultaneous size and quantitative mass profiling of Au NPs in liquor with online pre-channel calibration. To the best of our knowledge, there has been no report on the analysis of polydispersed Au NPs present in pristine food additive E175 and in E175-containing liquor by using the proposed approach.

## 2. Results and Discussion

### 2.1. Investigation of the Effects of Ethanol on Au NPs

There has been previous research [27,28] suggesting that a spontaneous linear aggregation of Au NPs occurred in ethanolic solution and the dipole–dipole interaction caused by the asymmetrical distribution of charges on the particle surfaces was assumed as the driving force for this aggregation. Aggregation is observed as a common transformation that changes the size and surface chemistry of nano-sized particles leading to alterations in their cellular uptake, toxicity, and overall fate [33]. It is thus important to determine and quantify the potential Au NP aggregation characteristics in gold-containing liquor in order for its comprehensive risk assessment.

To evaluate the effects of ethanol on the stabilization of Au NPs in alcoholic environments and their fractionation in AF4-ICP-MS analysis, various studies have been explored.

At first, to validate the hydrodynamic diameters (Dh) of the commercial Au NP standards, all the gold nanosphere standards were analyzed by DLS. As shown in Table 1, the Dh expressed as the Z-average diameter of each Au NP standard was obtained. These results complied well with the values declared by the manufacturer. Meanwhile, their particle size distributions were found to be unimodal, with the polydispersity indexes (PDI) ranging over 0.063–0.311. It suggested that these Au NP standards had highly monodispersed hydrodynamic sizes. Since the Dh value of 5 nm sized Au NPs was absent in its certificate, the in-house measured hydrodynamic diameters (i.e., Z-average diameter) of all Au NPs standards were adopted for size calibration in the following works.

**Figure 1:**
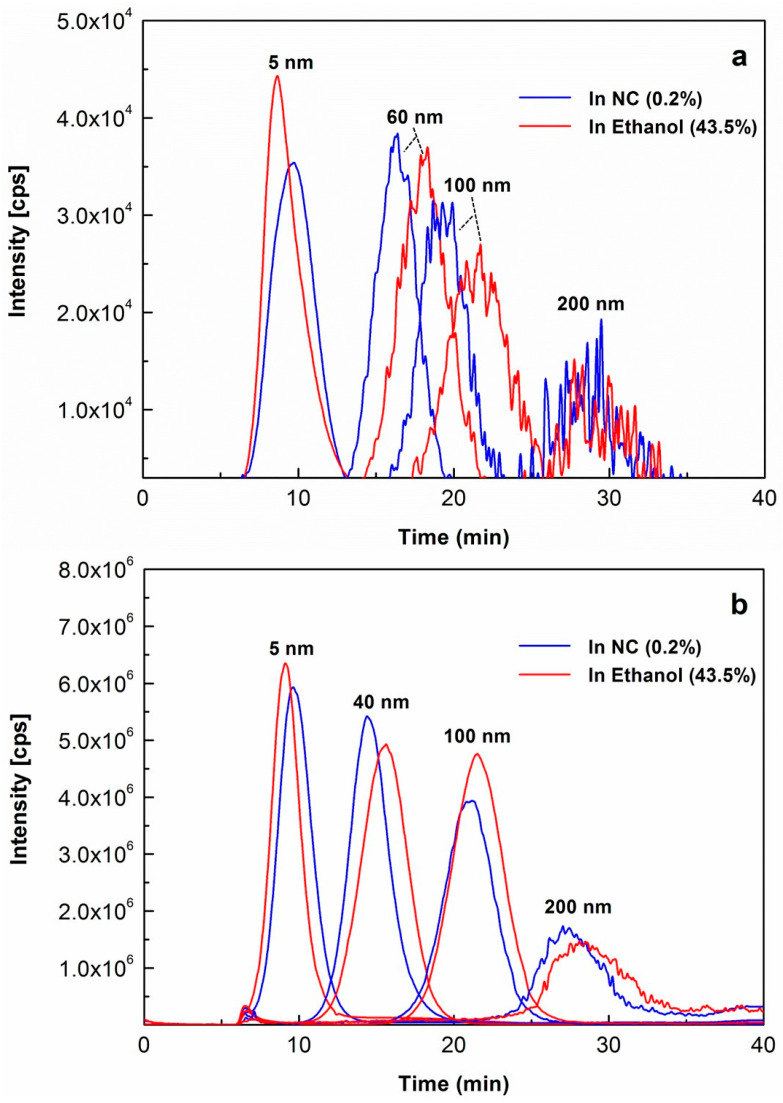

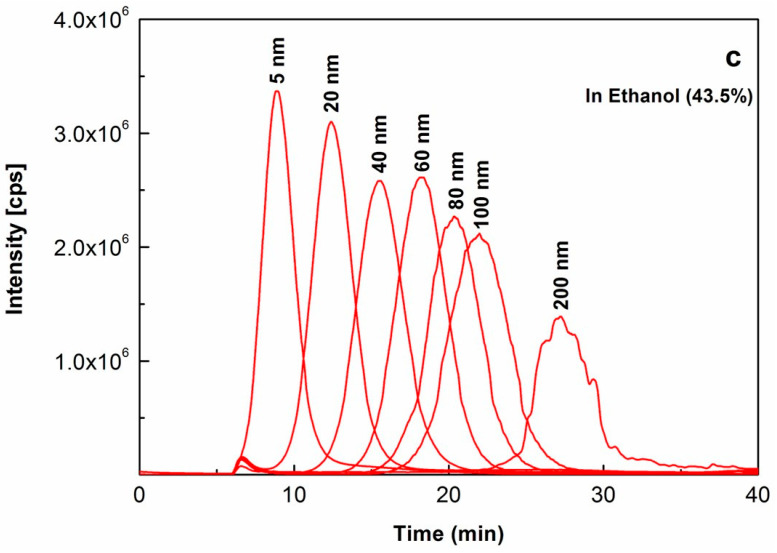
Effects of ethanol on stabilization of Au NPs in alcoholic suspensions and their separation for AF4-ICP-MS analysis, demonstrated by the overlaid fractograms of typical Au NPs size standards for comparisons under different solution environments and varied Au NPs concentrations, including in 0.2% NC vs. 43.5% ethanol at individual concentrations of (**a**) 50 µg L^−1^ or (**b**) 10,000 µg L^−1^, as well as (**c**) in 43.5% ethanol at 5000 µg L^−1^ for each of spiked Au NPs with wider size range.

Then, as shown in Figure 1a,b, the fractograms of typical Au NP size standards (5, 40, 60, 100 and 200 nm) under the same conditions of AF4-ICP-MS were separately overlaid for comparisons under different solvent environments and varied Au NPs concentrations, including in 0.2% NC vs. 43.5% ethanol at concentrations of 50 µg L^−1^ (Figure 1a) or 10,000 µg L^−1^ (Figure 1b). It was demonstrated that all peak maxima were well separated at these two concentrations with huge dynamic range (i.e., 50 µg L^−1^ (50 ppb)–10,000 µg L^−1^ (10 ppm)). No aggregation of Au NPs was observed in 43.5% ethanol all the time. Since there was no salt in all the investigated suspensions (i.e., 43.5% ethanol only), this observation of no aggregation of Au NPs occurring in 43.5% ethanol complied with the other previous finding that Au NPs can be stably dispersed in an ethanol suspension in the absence of salt [34]. It was reported in this previous paper that residual salt in combination with ethanol, instead of ethanol itself, induced the assembly of Au NPs in ethanol.

To affirm the stabilization of Au NPs in ethanolic dispersions and explore the size calibration of Au NPs based on their relevant AF4 fractionation behavior, Au NP standards with wider size coverage (5, 20, 40, 60, 80, 100 and 200 nm) spiked in 43.5% ethanol at 5000 µg L^−1^ were injected for AF4-ICP-MS analysis, respectively, analysis and their fractograms were overlaid as shown in Figure 1c. The concentration of 5000 µg L^−1^ was adopted, representing the typical concentration levels of gold (E 175) used in the exposure scenarios [1]. Again, all peak maxima were well separated. By plotting the in-house measured Dh values of the Au NP standards against the corresponding retention times of peak maxima (Relative Standard Deviation (RSD) ranging over 0.3–3.1%, n = 3 for each Au NP standard), the size calibration curve was then established. The modeling equation was established, i.e., y = 0.4581x^2^ − 6.4821x + 32.26 (y is the Dh in nm, x is Rt in min, R^2^ = 0.9984). Since the parabolic flow velocity profiles are generally present in the AF4’s channel, it was reasonably expected and observed that the regression between retention times and hydrodynamic sizes represented an excellent polynomial fit.

### 2.2. Determination of Au NPs Released after Ultrasound Treatment in Gold-Containing Liquor by AF4-ICP-MS

**Figure 2:**
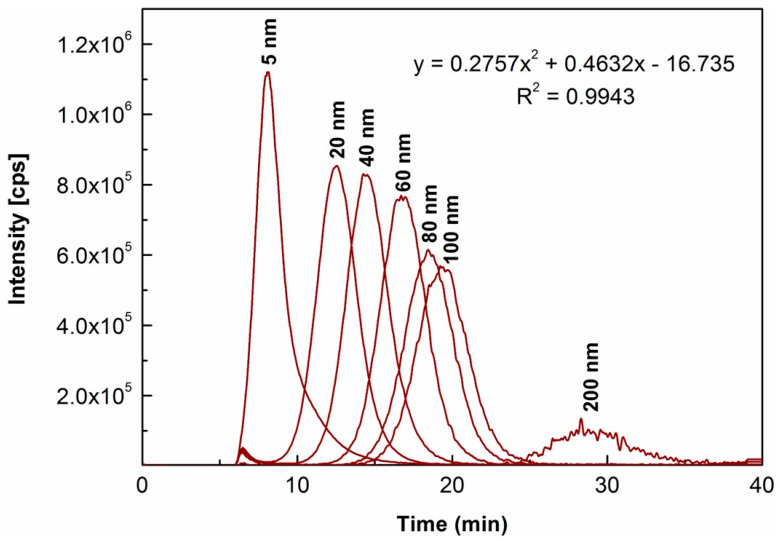
Overlaid AF4-ICP-MS fractograms of Au NPs standards dispersions (size-ladder) spiked in the blank liquor matrix (at 1000 µg L^−1^ for each), with the modeling equation displayed from corresponding size calibration curve (i.e., retention times of the AF4 peak maxima (min) (x) vs. nanoparticle diameters (nm) (y)).

In view that no extra aggregation of Au NPs occurred due to the effects in suspensions of 43.5% ethanol without salt in current work, and to further explore the effects of the matrix in the gold-containing liquor on size characterization of Au NPs, a series of Au NPs standards (5, 20, 40, 60, 80, 100 and 200 nm) spiked in the blank liquor matrix (at 1000 µg L^−1^ for each), as per the protocol described in Section 3.4, were analyzed under the same conditions. It aimed to establish the method with more sensitive detection of Au NPs at concentrations much lower than the commonly exposed levels of gold. As shown in Figure 2, their fractogram peaks were generally well resolved, thus allowing the size attribution of Au NPs in the unknown liquor samples. It was also worth noting that AF4 peaks of Au NP standards spiked in the blank liquor matrix were generally eluted a bit earlier than those of their counterparts in 43.5% ethanol (as shown in Figure 1c). Similar to the finding in a previous report [35], this variation in the fractionation behavior might result from the interferences from large particles of the ingredients (e.g., flavors of herbs and fruits) as the matrix in the gold-containing liquor together with the separation features of AF4. In the same way as above, the size calibration curve was also established, with Dh values plotted against the corresponding retention times of peak maxima (RSD ranging over 0.5–2.2%, n = 3). The modeling equation was y = 0.2757x^2^ + 0.4632x − 16.735 (R^2^ = 0.9943) and hereby used for size characterization of Au NPs in liquor.

#### 2.2.1. Protocol Optimization for the Sample Preparation

It is well known that, to conduct effective size characterization and mass quantification, nanoparticles such as Au NPs should be properly dispersed in a liquid medium. Since there was no aggregation of Au NPs due to the effects of ethanol and salt in the current studies, samples of the original liquor were initially prepared following the general workflows reported as before [35,36,37], such as shaking for homogenization and gentle sonication in a water bath. It was expected to easily detect and analyze Au NPs in the samples, in view of the unique features of gold such as chemically inert in bulk form, high atomic weight, and the low abundance of background in nature yielding few interfering signals. However, for all the samples prepared from the original liquor with the concentration of gold flakes at about 13 mg in one liter, regardless of sonication or not, there was no detection of Au NPs by AF4-ICP-MS analysis. This finding was in agreement with observations in another report [38] in which there were no Au NPs detected in pristine E175 products and E175-containing foods (e.g., liquors, wine confit, and syrup), based on the application of TEM and spICP-MS.

Considering the rationally expected presence of Au NPs at trace levels in E175 and relevant foods as well as the very low solubility of elemental gold, it was necessary to explore the optimized sample preparation method for size characterization and mass determination of Au NPs in the current study. In this regard, exploration for the enrichment of Au NPs in original gold-containing liquor and the subsequent ultrasound processing was conducted, aiming to achieve the effective detection of Au NPs in the samples.

**Figure 3:**
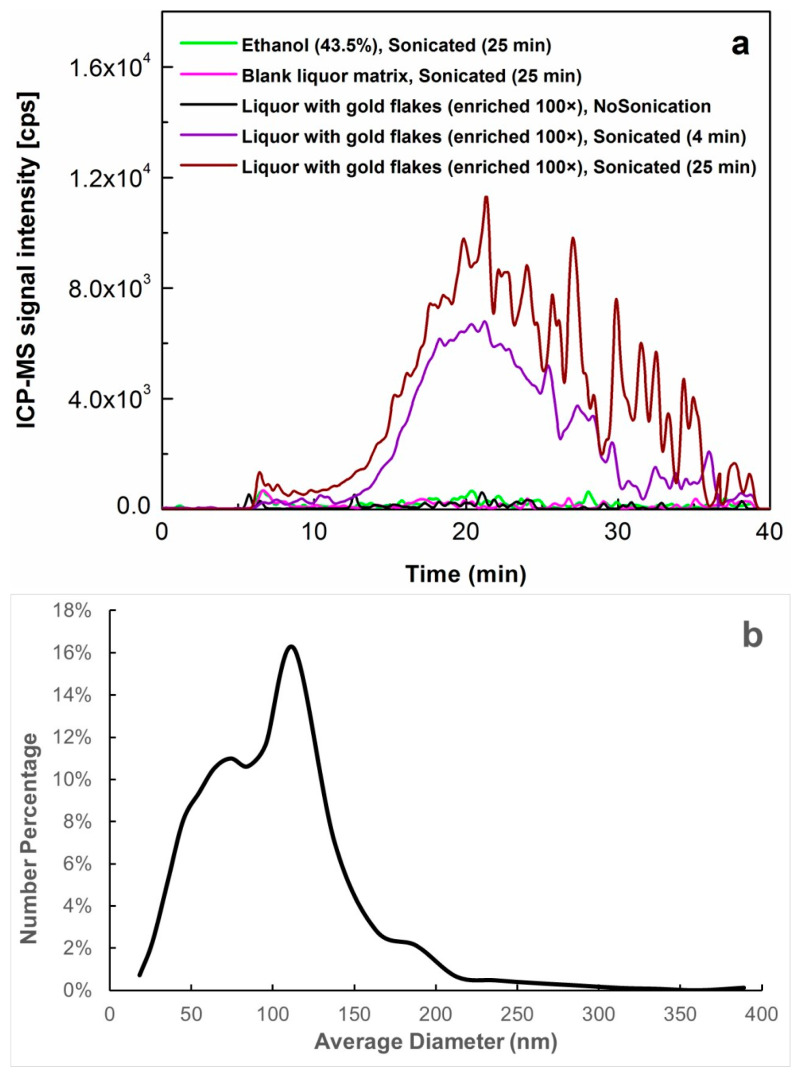
Profiling and characterization of the Au NPs with 100-fold enrichment and subsequent release in the studied gold-containing liquor after varied treatments of ultrasonic processing. (**a**) Overlaid AF4-ICP-MS fractograms of Au NPs in the processed liquor products and its blank liquor matrix under different scenarios, with the comparison of other blank control samples included as well. (**b**) Number-based size distribution of Au NPs detected in the concentrated gold-containing liquor based on ultrasound process for 25 min, as determined with AF4-ICP-MS and the following transformation of the mass-based to number-based distribution.

Figure 3a shows the overlaid AF4-ICP-MS fractograms of Au NPs in the processed liquor products and its blank liquor matrix under different scenarios. Optimization of the sample preparation method is briefed below. Firstly, although membrane-based filtration had been used for extraction and enrichment of Au NPs by other researchers [39], it was not adopted here due to the possible loss of gold flakes and the mass uncertainties introduced which may cause large inaccuracy in measurement. Instead, centrifugation for in-solution enrichment of gold flakes at low speed was applied, with the gold flakes concentrated and the potentially blank liquor matrix obtained. Based on the comparison of obtained results, the 100-fold enrichment was finalized enabling sufficient detection of Au NPs as discussed in the later sections. Secondly, to further extract Au NPs, ultrasound treatment of the concentrated gold flakes was investigated. In general, the most important parameters to be controlled during ultrasonication are sonication power and time because they may impose crucial effects on the properties of the Au NPs released. For this study, the constant settings of the ultrasonic water bath were applied all the time, i.e., a sonication frequency of 37 kHz and an effective power of 800 W, while the residence time for sonication varied from 0–25 min. As shown in Figure 3a, for the liquor with gold flakes enriched 100 times, when no sonication was applied, the fractogram was only detected as a baseline, i.e., no Au NPs detected; when the sonication time was increased up to 4 min, there was a great increase in the release and extraction efficiency of Au NPs; furthermore, a mild increase in the release and extraction of Au NPs into the liquor matrix between 4 min and 25 min was observed. This change of tendency in the extraction and detection of Au NPs was supportive of the assumption that the Au NPs were more likely to be present as nanoparticles in the forms of agglomerates and/or aggregates in the original gold flakes and then released off from micrometer-sized flakes under the ultrasound treatment. Otherwise, if Au NPs were mainly generated from the disruption of gold flakes by ultrasonication, significantly more Au NPs were expected to be detected with roughly parallel increments of signal intensity/peak area due to the extension of sonication time from 4 min to 25 min. It thus ensured the assumption that most/all of the available Au NPs should have been released into the liquor matrix. Hence, the optimized release and extraction time of Au NPs from gold flakes into the liquor matrix under ultrasound processing was finalized at 25 min. To serve as a blank control group, both the blank liquor matrix and the solvent of 43.5% ethanol were processed following the same workflow for sample preparation and subjected to ultrasound treatment for 25 min prior to AF4-ICP-MS under the same conditions. As shown in Figure 3a, their fractograms were also present as baseline. All these efforts enabled the favorable method for the release and extraction of Au NPs from the gold-containing liquor with 100-fold enrichment followed by ultrasound processing for 25 min.

#### 2.2.2. Characterization of Size and Number-Based Size Distribution of Au NPs

Subsequently, based on AF4-ICP-MS analysis of the released and extracted Au NPs in liquor with 100-fold enrichment of gold flakes and subsequent 25 min sonication (fractogram shown in Figure 3a), size characterization of the detected Au NPs was further conducted by using the abovementioned modeling equation established with Au NPs standards spiked in the blank liquor matrix. As summarized in Table 2, the Dh around 123.7 nm was determined as the dominant particle size of the released Au NPs in the liquor extract, using the retention time of peak maximum measured by AF4. The nano-size distribution range of the Au NPs was estimated to be around 8.3–398.0 nm, based on modeling calculation with the starting and ending retention times of the detected Au NPs over the range of 8.7–38.0 min.

To validate the established method and conduct independent verification of the characterized diameters of Au NPs released in liquor, as described in Section 3.3, the released and extracted Au NPs in a sample of the studied EGF product, with the concentration of about 1 mg EGF in 1 mL 43.5% ethanol and the treatment of 25 min sonication, was obtained and subjected to AF4-ICP-MS analysis using the same experimental conditions. The relevant size characterization of the released and extracted Au NPs in EGF was further conducted based on the modeling equation created with Au NPs size standards spiked in 43.5% ethanol, as described in Section 2.1. As shown in Table 2, the dominant Dh of Au NPs released in the EGF extract was determined around 126.6 nm, while the detected Au NPs inside ranged from 17.9 to 454.8 nm. In general, the released and extracted Au NPs in liquor and EGF had similar size profiles and closely characterized diameters.

To achieve the complementary profiling of the Au NPs released in the studied liquor, their number-based size distribution was also characterized with the previously proposed method [36], i.e., by conducting the transformation from mass-based signal and size distribution measured in AF4-ICP-MS to particle number-based size distribution. As shown in Figure 3b, the particle number-based size distribution of Au NPs released in the liquor with 100-fold enrichment and 25 min sonication was obtained from the mathematical conversion of mass-based AF4-ICP-MS fractogram. Apexes for the determined Dh in the size distribution curve were found around 112.9, 74.2, and 175.5 nm, which generally agreed with the mass-based fractogram pattern in AF4-ICP-MS analysis (Figure 3a). As per the number-based cumulative distribution, there was more than 50% (in number), i.e., 69.6%, of gold particles with diameters < 100 nm released and detected in the studied liquor.

As per the recommendation from EFSA [1], for E175 consumed in the powder form, its specifications about gold particles should be included regarding the mean particle size and particle size distribution, as well as the percentage (in number) of particles in the nanoscale present inside. The explorations herein for size characterization of Au NPs and determination of their number-based size distribution using AF4-ICP-MS provided an alternative solution in response to this requirement with some advantages in the current work. Firstly, the gold flakes in the original liquor were concentrated using low-speed centrifugation, and ultrasound processing was applied thereafter to enhance the release of Au NPs, both of which enabled better and more sensitive detection of Au NPs. Secondly, there was enrichment and fractionation of Au NPs by AF4 which further contributed to the increment of detected signal of Au NPs. In particular, based on comparison with some inherent challenges of the commonly used techniques, such as the ones from TEM including the costly price of equipment, low throughput efficiency, and requirement of highly specialized technical support, as well as the insufficiency of spICP-MS with unfavorable size detection limit of Au NPs, AF4-ICP-MS in this work is economical and practical for the routine analysis of Au NPs.

#### 2.2.3. Mass Quantification of Au NPs with Pre-Channel Mass Calibration

Compared with post-channel mass calibration, pre-channel mass calibration with suitable nanoparticle standards was expected to be more suitable for quantification and thus had been frequently used. It was probably because samples and all calibrants undergo the same procedure of injection and separation and the potential sample material loss was normalized [35,40]. Accordingly, the pre-channel mass calibration was adopted for mass quantification of Au NPs released in gold-containing liquor.

As mentioned in the above sections, the diameter of the prevalently released Au NPs was measured to be around 123.7 nm in the form of Dh rather than a physical diameter. Since Dh is deemed slightly larger than the nominal size, the Au NPs standard with a nominal size of 100 nm was used for the pre-channel mass calibration and subsequent quantification of total Au NPs inside. Using this approach, as shown in Table 3, based on 25 min ultrasonication, for the dominant Au NPs released with a nano-size range of 8.3–398.0 nm and centered around 123.7 nm, their mass content was measured as 48.1 μg L^−1^ in the liquor sample containing 100-fold concentrated gold flakes. For the gold-containing samples in the group as comparison control without ultrasonication, there was no detection of Au NPs, even for the samples with 100-fold enrichment. To evaluate the background noise from sample preparation and calibration, the blank control sample without any gold-containing liquor was quantified in parallel in the same way as above and only a negligible background signal was acquired. After subtraction of the background value, the Au NP concentrations as above were obtained. It was noted that, based on the above processing and measurement of Au NPs in liquor, even if all the detected Au NPs came from the disruption of gold flakes by ultrasonication, only 0.0037% of the mass content of gold was “transformed” from original flakes into Au NPs by ultrasonic energy. This very negligible rate further indicated the inappreciable possibility of direct generation of Au NPs from the disruption of gold flakes by ultrasonication.

In the meantime, the limit of detection (LOD) and limit of quantification (LOQ) in the current AF4-ICP-MS analysis were also evaluated. As reported previously [40] and proved by results in this work, the ICP-MS signal is proportional to the mass and mostly independent of the size of Au NPs. Table 3 shows the detection and quantification limits of Au NPs spiked in the blank liquor matrix. The LOD was found to be 1.4 μg L^−1^ and the LOQ was measured as 3.7 μg L^−1^, respectively. In general, these limits determined herein were comparable with previous reports by other researchers around 5 μg L^−1^ [41,42]. The presumable mass concentration of Au NPs was calculated as 0.5 μg L^−1^ (i.e., ~48.1 μg L^−1^/100) in the original liquor without 100-fold enrichment but with ultrasound treatment. This level of 0.5 μg L^−1^ was below the LOD of 1.4 μg L^−1^ for Au NPs, and it was reasonably understood that there was no detection of Au NPs in the sonicated original liquor samples. It thus implied that the enrichment of Au NPs was essential for its sensitive detection with sufficient abundance by AF4-ICP-MS.

Although this established method using AF4-ICP-MS is suitable for analysis of the liquid diet or beverages based on the studied model of gold-containing liquor, it is expected that, subjected to the necessary modifications, its application scope in this study can be extended to cover other similar foodstuffs containing gold flakes, e.g., solid foods like chocolates, confectioneries, and cake sprinkles. In general, the modifications should be conducted as per the ingredients of foods, focusing on the optimization of the early stage of sample preparation (e.g., selection of extraction solvent, experimental conditions for defatting if necessary). After the raw extracts of gold flakes from foods are obtained, the subsequent procedures for sample preparation and analysis of Au NPs can follow the workflow established in this study, such as centrifugation for enrichment of gold flakes, ultrasonication to release Au NPs, size characterization and mass determination of Au NPs with pre-channel calibration by AF4-ICP-MS analysis. The minor adjustment of certain experimental parameters may also be necessary according to the variations of foods.

### 2.3. Measurement of the Recovery in Au NPs Detection by AF4-ICP-MS with Pre-Channel Calibration

Regarding the mass measurement of Au NPs by using pre-channel calibration, their recovery rates were evaluated as well. Pre-channel calibration with Au NPs standards compensated for the possible losses of sample materials resulting from interactions between Au NPs and the parts in the AF4 channel such as the permeation membrane and other surfaces of parts. The interactions were normally the major source of low recoveries. Moreover, the extraction efficiency of Au NPs and the interference by the food matrix also had to be considered in the samples. Hence, the losses of sample materials were studied by individual use of Au NP dispersions with nominal diameters of 5, 20, 60, 100 and 200 nm. As shown in Table 4, the overall recoveries (R) were satisfactory for all five sizes of Au NPs ranging over 82–95%. Although the recovery rate for 5 nm particles was slightly lower than others, all the recoveries in Table 4 indicated that there was no obvious loss of Au NPs coming from either the interference by the ultrasound processing, the extraction from the liquor matrix or the application of cross flow in AF4 separation. It suggested that the established pre-channel mass calibration worked sufficiently.

## 3. Materials and Methods

### 3.1. Instrumentation

For the AF4-ICP-MS analysis, instrumental conditions similar to those previously reported in the internal lab work [35,36,37] were applied. Briefly, an Agilent 7900 ICP-MS system containing a Micromist nebulizer and a Scott-type spray chamber (Santa Clara, CA, USA) was utilized under the optimized conditions. Instrument tuning was conducted before analysis, aiming to obtain high detection sensitivity and reduce the interfering signals from the background. The ICP-MS parameters applied were as follows: RF power, 1550 W; plasma gas (Ar), 15.0 L min^−1^; carrier gas (Ar), 1.09 L min^−1^; auxiliary gas (Ar), 0.9 L min^−1^. The ICP-MS data acquisition was conducted with Time Resolve Analysis (TRA) in He mode and the isotope of *m*/*z* 197 for gold was monitored with an integration time of 0.5 s.

For evaluation of size distribution of Au NPs standards in the form of Dh, the DLS measurements were performed in disposable polystyrene cuvettes at 25 °C using a Zetasizer Nano ZS (Malvern Instruments, Malvern, UK), with a refractive index of 1.330 and a viscosity of 0.8872 cp to mimic the values of ultrapure water.

The hyphenated AF4-ICP-MS system containing an AF2000 instrument from Postnova Analytics (Landsberg am Lech, Germany) coupled to the Agilent 7900 ICP-MS was applied for Au NPs analysis. A 350 μm spacer and 10 kDa regenerated cellulose membrane were utilized for the AF4 separation. Au NPs separation was performed using NovaChem Surfactant 100 (NC), a commercial product of surfactant mixture comprising non-ionic and ionic detergents, at its concentration of 0.05% as the AF4 carrier. The conditions used for AF4 separation were as follows. A constant cross flow of 1 mL min^−1^ was initially applied, with the sample injection volume at 20 μL. After 4 min for sample injection and 1 min for focusing and transitioning, the cross flow was decreased in a power manner (exponent 0.2) to 0 mL min^−1^ over a period of 33 min. The tip flow was also decreased until a flow rate of 0.5 mL min^−1^ was reached and kept constant for continuous running, leading to a total AF4 running time of 40 min. A constant detector flow rate was kept at 0.5 mL min^−1^ throughout all the steps of injection, focusing, and separation.

### 3.2. Reagents and Materials

The product of NovaChem Surfactant 100 (NC) was obtained from Postnova Analytics (Landsberg am Lech, Germany). Absolute ethanol (ACS reagent grade, ≥99.9%) was obtained from Merck (Darmstadt, Germany). The working buffers of 0.2% NC and 43.5% (*v*/*v*) ethanol were freshly prepared. Milli-Q ultrapure water was used for all the works.

Gold Nanosphere dispersions (NanoXact) with 5, 20, 40, 60, 80, and 100 nm nominal diameters were obtained from nanoComposix (San Diego, CA, USA). All standards were supplied as suspensions of Au NPs at 50 mg L^−1^ and stabilized in the citrate buffer. Au NPs standard (Product Number: 742066) with a nominal diameter of 200 nm was from Sigma-Aldrich (St. Louis, MO, USA). This Au NPs standard was also a stabilized suspension in citrate buffer with a particle number concentration of approximately 1.9 × 10^9^ particles mL^−1^. Suspensions of these Au NP size standards were individually diluted in a suitable buffer (e.g., 0.2% NC or 43.5% ethanol) prior to instrumental analysis.

A commercial liquor containing free-floating thin and visible gold flakes (~13 mg in a 1 liter bottle), as well as 43.5% alcohol (by volume), was purchased from a local distributor. One commercial edible gold flakes product (EGF) was also purchased in a local market as the reference additive.

### 3.3. Sample Preparation for Release and Extraction of Au NPs

To release and extract Au NPs for characterization and determination, the commercial liquor and the individual edible gold flakes product (EGF) were separately processed based on a similar ultrasound treatment. Briefly, the liquor was thoroughly shaken for homogenized distribution of the free-floating gold flakes inside. About 100 mL of liquor sample was measured followed by the subsequent centrifugation at low speed (3000 rpm (2113× *g*), 5 min). Then, about 99 mL of the upper phase/supernatant as a clear liquor matrix was carefully removed and collected, yielding about 1 mL of the remaining liquor suspension of visible gold flakes. Both of about 1 mL of these two liquor layers obtained, i.e., the blank matrix at the upper phase and the concentrated suspension of gold flakes at the bottom phase, were separately measured for their individual volumes, vortexed for 30 s, and were/were not subjected to the subsequent ultrasonication using an ultrasonic water bath (FisherBrand, model: FB 15065) from Fisher Scientific (Loughborough, Leicestershire, UK). The non-ultrasound-treated counterparts of these two liquor layers were used as controls, respectively. The ultrasound process was conducted at a sonication frequency of 37 kHz and an effective power of 800 W for 4 min and 25 min, respectively, with the temperature controlled at around 25 °C using an ice bag to prevent the samples from being heated up due to the ultrasound energy. The samples were then vortexed for 30 s prior to injection for AF4-ICP-MS analysis. Similarly, about 1 mg of the EGF product was weighed and mixed with 1 mL of 43.5% ethanol. The obtained alcoholic suspension of gold flakes of EGF was separately subjected to ultrasound-assisted release and extraction of Au NPs as above protocols for AF4-ICP-MS analysis as well.

### 3.4. Size Characterization of Au NPs by AF4-ICP-MS

To normalize the interfering effects and characterize the size of Au NPs potentially released in the commercial gold-containing liquor, the blank liquor matrix was obtained with sonication for 25 min as per protocols in Section 3.3, individually spiked with a series of Au NP standards (5, 20, 40, 60, 80, 100 and 200 nm), and yielding the corresponding size ladder suspensions (1 mL at 1000 μg L^−1^ for each). Furthermore, to verify the presence of Au NPs presumably released from EGF and characterize their sizes, 43.5% ethanol was applied to replace the blank liquor matrix for preparation of the same Au NP size ladder suspensions as above under the same experimental conditions and procedures. These Au NP standards suspensions in liquor matrix or 43.5% ethanol were further injected for AF4-ICP-MS analysis, respectively, under the same conditions. By plotting the in-house measured values of Dh of all Au NPs standards against corresponding elution times at peak maxima in AF4 fractograms, a regression equation could be established for modeling the relationship between Rt and Dh through the polynomial interpolation processing function. Thus, the size of Au NPs in samples can be characterized.

### 3.5. Quantification of Au NPs by Pre-Channel Mass Calibration

To quantify the mass content of Au NPs present in the gold-containing liquor, pre-channel calibration using Au NP standards was conducted. In short, the blank liquor matrix obtained with sonication for 25 min was spiked with Au NP standard (100 nm) stock suspension in 7 centrifuge tubes to obtain a series of standard suspensions at their final concentrations of 1, 2, 5, 10, 20, 50 and 100 µg L^−1^. By plotting these spiked concentrations against the corresponding peak areas obtained by pre-channel injection and online AF4-ICP-MS analysis, the samples of concentrated gold-containing liquor that were not treated/treated by sonication for 25 min were analyzed to quantify the present Au NPs.

### 3.6. Measurement of Limit of Detection (LOD) and Limit of Quantification (LOQ)

As per the protocol in Section 3.5, the dispersions of Au NPs standard (e.g., 100 nm) were prepared in liquor matrix with decreasing concentrations (ranging over 100–1 µg L^−1^) and injected for AF4-ICP-MS analysis. The LOD was defined as the lowest concentration of the Au NPs in liquor matrix that can be detected with the minimum signal-to-noise ratio of 3:1. The LOQ was defined as the lowest concentration of the Au NPs in liquor matrix that can be quantified with the minimum of signal-to-noise ratio of 10:1.

### 3.7. Evaluation of Recovery Rates in Au NPs Analysis by AF4-ICP-MS

To conduct the recovery experiments, all possible factors accounting for total losses of Au NPs in the sample need to be covered, such as the interference of liquor matrix, and the effects of interactions between Au NPs and membrane or other surfaces in the AF4 system. The same internal methods and equations previously reported [35] for the analysis of recovery rates were also applied. In brief, for the measurement of Recovery (R_A_) of Au NPs due to its release from the gold flakes in the gold-containing liquor and the interference of liquor matrix, the selected Au NP standards spiked in blank liquor matrix (i.e., 5, 20, 60, 100 and 200 nm, 50 µg L^−1^ for each of sizes at and below 100 nm, 100 µg L^−1^ for 200 nm) were injected for AF4-ICP-MS analysis. The resultant ICP-MS peak areas of ^197^ Au were compared and R_A_ was expressed as R_A_ (%) = [(A_2_ − A_1_)/A] × 100, where A_2_, A_1_, and A represented the peak areas obtained in the analysis of spiked and un-spiked liquor matrices, as well as those of the same Au NPs standard spiked in 0.2% NC, respectively, under the normal cross flow in AF4 separation. For the determination of recovery (R_B_) caused by the interactions of Au NPs with the membrane or other surfaces in the AF4 channel, the same selected Au NP standards as above were spiked in 0.2% NC and injected for AF4-ICP-MS analysis, respectively. Accordingly, R_B_ was calculated by R_B_ (%) = (B/B_0_) × 100, where B and B_0_ were peak areas of the same Au NP standard separately obtained with or without cross flow applied in AF4 separation. Based on the integration of R_A_ and R_B_, the overall recovery rate (R) can be defined and calculated as R (%) = [(A_2_ − A_1_)/B_0_] × 100.

## 4. Conclusions

In this study, an effective and pragmatic method using AF4-ICP-MS has been newly established for the size and mass profiling of Au NPs released in gold-containing liquor with the ultrasound treatment. Additionally, AF4-ICP-MS analysis of EGF as the pristine food additive E175 under the same conditions further validated the efficiency of the established method and supported the characterization of diameters and particle size distribution of Au NPs released in liquor. It is worth noting that the established method in this study is normally applied to the analysis of spherical/quasi-spherical Au NPs based on the size and mass calibration with a series of gold nanosphere standards. The enhanced characterization of the non-spherical Au NPs will be further explored in the next stage of relevant works.

In summary, the AF4-ICP-MS method developed herein enabled effective and economical monitoring of the potential presence of Au NPs in edible gold-containing liquor and the gold food additive (E175), especially serving as an alternative solution in response to EFSA’s recommendation for size characterization and number-based size distribution/profiling of Au NPs present in the powder form of gold. It is believed that subject to suitable modifications, this method could also be applicable to other gold-containing foods under common food manufacturing conditions.

## Figures and Tables

**Table 1:** Measurement and assessment of the commercial gold nanosphere dispersions used for size characterization.

Size Reported in Product Certificate ^a^			DLS Analysis Value ^b^ (nm)	
Normal Size (nm)	TEM Diameter (nm)	Hydrodynamic Diameter (DLS) (Dh) (nm)	Z-Average Diameter (nm)	PDI
5	5.0 ± 0.6	─ ^c^	10.1 ± 0.5	0.311 ± 0.034
20	18.9 ± 1.5	24.0	22.4 ± 0.1	0.141 ± 0.002
40	40.0 ± 5.0	45.0	43.1 ± 0.3	0.113 ± 0.010
60	60.0 ± 6.0	68.0	70.2 ± 0.3	0.094 ± 0.013
80	77.0 ± 10.0	83.0	83.3 ± 0.3	0.158 ± 0.013
100	103.0 ± 10.0	105.0	110.5 ± 0.2	0.063 ± 0.007
200	200.0	213.0	212.9 ± 3.3	0.097 ± 0.019

^a^ Size information as listed in the manufacturer’s certificates. ^b^ Z-average diameter and polydispersity index (PDI) measured by in-house DLS analysis (n = 3). ^c^ Not available.

**Table 2:** Size characterization of Au NPs released by ultrasound treatment in edible gold flakes product and the concentrated gold-containing liquor investigated in this study (n = 3).

Product ^a^	Retention Time Range (min)	Hydrodynamic Diameter (Dh) (nm)	
		Major Diameter (nm) ^b^	Size Range (nm) ^c^
Gold-containing Liquor	(8.7 ± 0.2)~(38.0 ± 0.1)	123.7 ± 5.5	(8.3 ± 1.1)~(398.0 ± 2.7)
Edible Gold Flakes (EGF)	(11.4 ± 0.4)~(38.3 ± 0.4)	126.6 ± 9.8	(17.9 ± 1.4)~(454.8 ± 12.3)

^a^ Samples prepared for Au NP size characterization as per methods described in Section 3.3 of this study with the ultrasound process applied for 25 min. ^b^ Dominant Dh of Au NPs based on size calibration and retention time at predominant peak over the AF4 fractogram. ^c^ Based on size calibration and calculation over the period of starting and ending retention times of detected Au NPs in AF4 fractogram.

**Table 3:** Quantification and measurement of the limits of detection and quantification of Au NPs potentially released and present in gold-containing liquor using AF4-ICP-MS under the scenarios of ultrasound processing.

Mass Concentration of Au NPs in Liquor (μg L^−1^) ^a^		Limit of Detection (LOD) (µg L^−1^) ^d^	Limit of Quantification (LOQ) (µg L^−1^) ^d^
No Sonication	Sonication ^b^		
	Liquor with 100-Fold Enrichment		
No Detection	48.1 ± 0.6 ^c^	1.4	3.7

^a^ Based on detection of Au NPs in the prepared suspension without/with sonication enhancing gold nanoparticles release and the quantification by pre-channel calibration with Au NPs standard (100 nm) and AF4-ICP-MS analysis (n = 3). ^b^ Measurement based on the ultrasound process at a sonication frequency of 37 kHz and an effective power of 800 W for the maximum period of 25 min. ^c^ The mass concentration of Au NPs released in the liquor in which the content of gold flakes was enriched 100 fold, followed by the subsequent ultrasonication for 25 min. ^d^ Based on measurement of Au NPs standards with nominal diameters ranging over 5–200 nm spiked in the blank liquor matrix.

**Table 4:** Recovery rates measured by AF4-ICP-MS for Au NPs detected in the gold-containing liquor with pre-channel calibration (n = 3).

Nominal Size (nm)	Recovery Rate (R)— Overall (%)	Recovery Rate (R_A_)— Matrix (%)	Recovery Rate (R_B_)— AF4 Channel (%)
5	82 ± 2	89 ± 1	92 ± 2
20	93 ± 2	101 ± 3	93 ± 1
60	92 ± 7	94 ± 1	98 ± 7
100	95 ± 2	98 ± 1	97 ± 2
200	91 ± 2	97 ± 1	94 ± 2

## Data Availability

Data are contained within the article.
